# Interleukin-17 induced by cumulative mild stress promoted depression-like behaviors in young adult mice

**DOI:** 10.1186/s13041-020-00726-x

**Published:** 2021-01-13

**Authors:** Jinho Kim, Yoo-Hun Suh, Keun-A Chang

**Affiliations:** 1 Department of Health Sciences and Technology, GAIHST, Gachon University, Incheon, 21936 Korea; 2 Neuroscience Research Institute, Gachon University, Incheon, 21565 Korea; 3 Department of Pharmacology, College of Medicine, Gachon University, Incheon, 21936 Korea

**Keywords:** Cumulative mild stress, Brain development, Young adulthood, Depression-like behavior, Anxiety, Inflammation, Interleukin-17

## Abstract

The number of young adult patients with major depression, one of the most common mental disorders, is gradually increasing in modern society. Stressful experiences in early life are considered one of the risk factors for chronic depressive symptoms, along with an abnormal inflammatory response in later life. Although increased inflammatory activity has been identified in patients with depression, the cause of long-lasting depressive states is still unclear. To identify the effects of cumulative mild stress in brain development periods, we generated a young adult depression mouse model exposed to cumulative mild stress (CPMS; cumulative mild prenatal stress, mild maternal separation, and mild social defeat) to mimic early life adversities. CPMS mice exhibited more long-lasting anxiety and depression-like behaviors than groups exposed to single or double combinations of mild stress in young adult age. Using the molecular works, we found that inflammatory cytokines, especially interleukin (IL)-17, upregulated microglial activation in the hippocampus, amygdala, and prefrontal cortex of CPMS mice. In the brains of CPMS mice, we also identified changes in the T helper (Th)-17 cell population as well as differentiation. Finally, anti-IL-17 treatment rescued anxiety and depression-like behavior in CPMS mice. In conclusion, we found that cumulative mild stress promoted long-lasting depressive symptoms in CPMS mice through the upregulation of IL-17. We suggest that the CPMS model may be useful to study young adult depression and expect that IL-17 may be an important therapeutic target for depression in young adults.

## Introduction

Major depressive disorder (MDD) is a chronic psychiatric disease characterized by a long-lasting state of depressive symptoms such as anxiety, loss of pleasure, and a feeling of low self-worth according to the World Mental Health Survey version of the Composite International Diagnostic Interview [1–4]. Patients with early-onset depression often experience a loss of social relationships, suffer from physical health problems, and exhibit high-risk sexual behavior [5, 6]. Notably, young adult patients with major depression have experienced more depressive episodes and attempt suicide than those with late-onset MDD [7]. Moreover, the number of young adult patients with MDD is consistently increasing, and 2–12% of these have been reported to have previously attempted suicide [8, 9]. Stressful experiences during early life can be associated with psychiatric disorders such as MDD and post-traumatic stress disorder [10, 11]. Due to the rapid brain development and epigenetic regulation that occurs between childhood and adolescence, early life stress may negatively alter the brain’s connections and functions [12]. However, the underlying molecular mechanisms that evoke chronic depression are not clear.

Recent studies suggest that IL-17, a pro-inflammatory cytokine, is involved in the pathogenesis of several inflammatory diseases, autoimmune diseases, and MDD [13]. In clinical studies, adult patients with depression exhibited higher IL-17 levels in serum than in healthy controls and have more Th17 cells, which produce IL-17 [14, 15]. Previous studies have also shown increased Th17 cells in the brains of the learned helplessness rodent model [14, 16]. Th17 cells promote microglial activation, neuroinflammation, and neuronal damage [17]. Differentiation of Th17 cells from naïve CD4+ T helper cells requires the elevated expression of cytokines IL-6 or IL-21 which, in concert with transforming growth factor-beta (TGF-β), drive activation of signal transducer and activator of transcription 3 (STAT3) [18]. This transcription factor promotes the expression of RAR-related orphan receptor γt (RORγt) [19, 20]. Also, TGF-β indirectly promotes the differentiation of Th17 cells by preventing Th1 and Th2 cell differentiation [21–23].

Here, we hypothesized that cumulative mild stress during brain development is associated with abnormal regulation of IL-17 and may lead to long-lasting depression in young adults. To prove our assumption, we investigated whether cumulative mild stress during a critical developmental period induces chronic depression-like behavior in a mouse model. We also examined the effect of IL-17 on depression-like behavior to determine how it evokes chronic depressive symptoms.

## Results

### Cumulative mild stress led to depression-like behavior in young adult mice

**Fig. 1:**
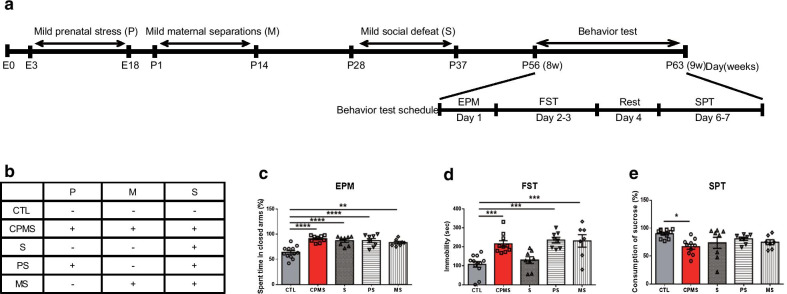
Cumulative mild stress in early life led to more severe anxiety- and depression-like behavior in young adults aged. **a** Experimental scheme included cumulative mild stress and behavior tests to evaluate the CPMS mice model. The mice fetuses were indirectly exposed to unpredictable mild stress (E3–18) through their dams and litters were directly exposed to mild maternal separation (P1–14) and mild social defeat stress (P28–37). **b** The table displayed each group exposed to mild stress. Following induction of stress, **c** EPM (CTL (n = 12); 63.75 ± 3.512, CPMS (n = 9); 90.89 ± 2.201, S (n = 8); 87.00 ± 3.333, PS (n = 8); 87.25 ± 4.178, MS (n = 7); 83.14 ± 2.650), **d** FST (CTL; 107.8 ± 15.06, CPMS; 216.4 ± 16.36, S; 130.0 ± 18.20, PS; 236.0 ± 14.89, MS; 231.7 ± 33.10), and **e** SPT (CTL; 89.91 ± 2.337, CPMS; 66.70 ± 4.549, S; 73.66 ± 9.951, PS; 80.63 ± 2.903, MS; 74.71 ± 4.460) were performed in 8-week-old mice. After the behavior tests, 9-week-old mice were sacrificed for molecular works

C57BL/6N mice were exposed to mild prenatal stress (P), mild maternal separation (M), and mild social defeat stress (S) in the growth process (Additional file 1: Fig. S1). To prove that cumulative mild stress in early life more affects long-lasting depressive symptoms, we compared the CPMS group with a single or double combination mild stress groups (Fig. 1a, b). In the elevated plus-maze (EPM) test, all stress groups showed higher levels of anxiety at 8 weeks compared with the CTL group (Fig. 1c, F_(4,39)_ = 12.80, *p* < 0.0001). Immobility time was significantly increased in the CPMS (*p* < 0.0020), PS (*p* < 0.0002), and MS (*p* < 0.0006) groups, but not in S group (Fig. 1d, F_(4,40)_ = 10.65, *p* < 0.0001). However, in the SPT, sucrose consumption was significantly reduced only in the CPMS group compared to the CTL group (Fig. 1e, F_(4,39)_ = 3.255, *p* = 0.0214).

### Anti-depressant treatment recovered anxiety and depression-like behavior in CPMS mice

**Fig. 2:**
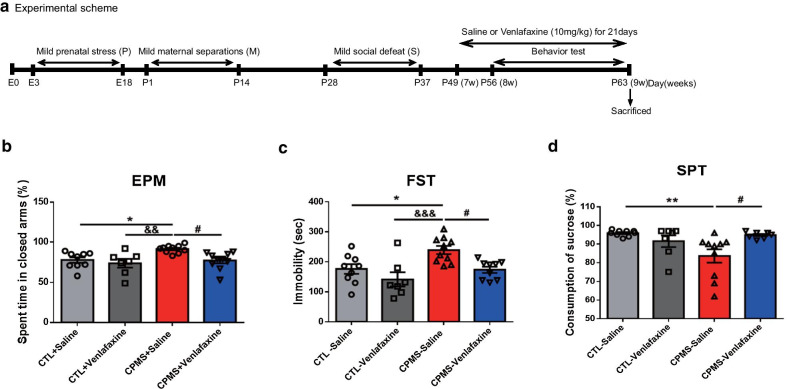
The anxiety- and depression-like behavior in CPMS mice were recovered by anti-depressant treatment. **a** 7-week-old mice were treated with venlafaxine or saline for 3 weeks until they were 9 weeks of age before the behavior test. **b** The anxiety level of each group was measured using duration time in closed arms in elevated plus maze (EPM) (CTL-saline (n = 9); 77.67 ± 3.399, CTL-venlafaxine (n = 7); 73.71 ± 5.366, CPMS-saline (n = 10); 91.40 ± 1.500, CPMS-venlafaxine (n = 10); 77.00 ± 3.370). **c** The immobility time of mice in the forced swim test (FST) was determined to measure despair (CTL-saline; 175.8 ± 16.33, CTL-venlafaxine; 141.4 ± 23.46, CPMS-saline; 239.2 ± 13.48, CPMS-venlafaxine; 173.0 ± 10.36). **d** Ratio of sucrose consumption in the sucrose preference test (SPT) was determined to measure anhedonia (CTL-saline; 95.89 ± 0.5386, CTL-Venlafaxine; 91.57 ± 3.146, CPMS-saline; 83.60 ± 3.603, CPMS-Venlafaxine; 94.88 ± 0.6105). All data are presented as mean ± SEM. *p < 0.05, **p < 0.01, vs. CTL-saline, ^&&^*p* < 0.01, ^&&&^*p* < 0.001 vs CTL-venlafaxine, ^#^*p* < 0.05 vs CPMS-venlafaxine

It was confirmed that the anxiety and depression-like behaviors in CPMS mice were recovered with antidepressant venlafaxine treatment. 8-week-old mice, who started antidepressant administration from 7 weeks of age, were examined for anxiety, despair, and anhedonia (Fig. 2a). The CPMS-saline group (*p* < 0.0346) had significantly higher levels of anxiety compared to the control (CTL) groups. However, venlafaxine treatment reduced anxiety levels in the CPMS group (Fig. 2b, F_(3,32)_ = 5.569, *p* < 0.0034). In the forced swimming test (FST), immobility time was increased in the CPMS-saline group (*p* < 0.0269) compared to CTL groups, and immobility was recovered in the CPMS-venlafaxine group (Fig. 2c, F_(3,31)_ = 6.950, *p* = 0.0010). Also, in the sucrose preference test (SPT), CPMS-saline mice showed reduced sucrose consumption compared to the CTL group (*p* = 0.0062 vs CTL-saline group). However, sucrose intake in CPMS-Venlafaxine mice was similar to that of the control-saline group (*p* = 0.0007 vs CPMS-saline group; Fig. 2d). There were no significant differences in movements of open field test (OFT) and memory of the Y-maze test (Additional file 1: Fig. S2).

### Cumulative mild stress upregulated inflammatory responses, especially IL-17, as well as microglial activation

After cumulative mild stress in early life, we observed the central or peripheral changes in the CPMS mice (Additional file 1: Fig. S3A). The bodyweight of the CPMS group was decreased compared to the CTL group (Additional file 1: Fig. S3B). Also, corticosterone in the serum of 8-week-old CPMS mice was significantly increased compared with CTL mice (Additional file 1: Fig. S3C).

**Fig. 3:**
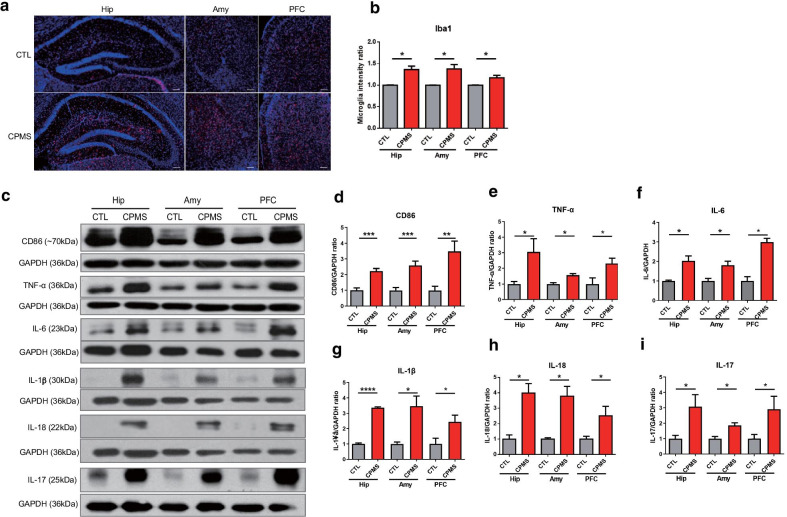
Cumulative mild stress induced increase of inflammatory cytokines and microglia activation in young adult CPMS mice. **a** Immunofluorescence staining for Iba1(red) was performed in the 8-week-old mice (n = 3 per group). scale bar 100 μm. **b** Microglia intensity was quantified in each region (Hip; 1.362 ± 0.08311, Amy; 1.377 ± 0.1037, PFC; 1.172 ± 0.05855). **c** Western blot analysis was performed using the cytosol isolated from hippocampus (Hip), amygdala (Amy) and prefrontal cortex (PFC). In each region, **d** CD86 (Hip; 2.227 ± 0.1656, Amy; 2.575 ± 0.2834, PFC; 3.479 ± 0.6652 vs CTL). **e** TNF-α (Hip; 3.054 ± 0.8463, Amy; 1.577 ± 0.09831, PFC; 2.311 ± 0.3439 vs CTL), **f** IL-6 (Hip; 1.850 ± 0.2804, Amy; 3.334 ± 0.8327, PFC; 1.923 ± 1.853 vs CTL), **g** IL-1β (Hip; 3.363 ± 0.05453, Amy; 3.459 ± 0.6625, PFC; 2.442 ± 0.4368 vs CTL), **h** IL-18 (Hip; 3.985 ± 0.6116, Amy; 2.511 ± 0.6282, PFC; 2.511 ± 0.6107 vs CTL), and **i** IL-17 (Hip; 3.082 ± 0.7783, Amy; 1.861 ± 0.1675, PFC; 2.908 ± 0.8383 vs CTL) were normalized by GAPDH. All data are presented as mean ± SEM of three independent experiments. *p < 0.05, **p < 0.01, ***p < 0.001 vs. CTL

Referring to previous studies that showed alterations in microglial activation and changes in frontolimbic volume in depression mouse models [24, 25], we focused on changes in the hippocampus (Hip), amygdala (Amy), and prefrontal cortex (PFC) of 8-week-old mice. We found an increase in the intensity of microglia in the Hip (*p* = 0.0123), Amy (*p* = 0.0221), and PFC (*p* = 0.0429) in the CPMS group compared to the CTL group (Fig. 3a, b). To confirm activated microglia, we also performed western blot analysis using CD86 as an activated microglia marker. The level of CD86 expression was significantly increased in the CPMS group compared with the CTL group (Hip, *p* = 0.0007; Amy, *p* = 0.0014; PFC, *p* = 0.0027; Fig. 3c, d and Additional file 1: Fig. S4A).

Next, we observed changes in the levels of important inflammatory factors, Tumor necrosis factor-alpha (TNF-α) and IL-6 in the brains of 8-week-old mice via western blot analysis. TNF-α (Hip, *p* = 0.0381; Amy, *p* = 0.0279; PFC; Fig. 3e and Additional file 1: Fig. S4B) and IL-6 (Hip, *p* = 0.0165; Amy, *p* = 0.0178; PFC, *p* = 0.0210; Fig. 3f and Additional file 1: Fig. S4C) in Hip, Amy, and PFC of CPMS group were significantly increased compared to the CTL group. Also, the level of IL-1β (Hip, *p* < 0.0001; Amy, *p* = 0.0266; PFC, *p* = 0.0469; Fig. 3g) and IL-18 (Hip, *p* = 0.0037; Amy, *p* = 0.0122; PFC, *p* = 0.0496; Fig. 3h) was also increased in the brain of CPMS mice. Especially, IL-17 (Hip, *p* = 0.0311; Amy, *p* = 0.0042; PFC, *p* = 0.0478; Fig. 3g and Additional file 1: Fig. S4C) in Hip, Amy, and PFC of the CPMS group were significantly increased compared to the CTL group. Besides, the levels of TNF-α, IL-6, and IL-17 in the serum of the CPMS group were also significantly increased compared to the CTL group (Additional file 1: Fig. S5). In our results, we confirmed that activated microglia and upregulated inflammatory responses persist in the young adult age of CPMS mice.

### Differentiation and population of Th17 cells was increased in the brain of CPMS mice

Because we discovered high levels of IL-17 in the brains and peripheral blood of CPMS mice, we have focused on the changes in the signaling pathway related to the differentiation of Th 17 cells.

**Fig. 4:**
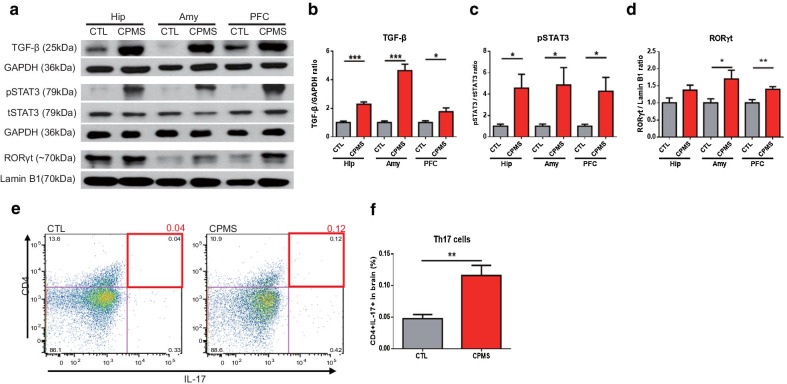
Differentiation and population of Th17 cells was increased in the brains of CPMS mice. **a** Representative image of western blot analysis in the Hip, Amy, and PFC. **b** TGF-β (Hip; 2.288 ± 0.1537, Amy; 4.342 ± 0.4557, PFC; 1.757 ± 0.2707 vs CTL), and **c** pSTAT3/tSTAT3 (Hip; 4.563 ± 1.284, Amy; 4.850 ± 1.628, PFC; 4.278 ± 1.152 vs CTL) were normalized by GAPDH. **d** RORγt (Hip; 1.374 ± 0.1462, Amy; 1.700 ± 0.2548, PFC; 1.399 ± 0.215 vs CTL) was normalized by Lamin B1. **e** Flow cytometry was performed to detect CD4^+^ IL-17^+^ cells (CTL (n = 5); 0.0476 ± 0.006592, CPMS (n = 6); 0.1158 ± 0.01638) in whole brain of the 8-week-old mice. All data are presented as mean ± SEM of three or more independent experiments. *p < 0.05, **p < 0.01, ***p < 0.001 vs. CTL

At first, we observed differentiation factors required for development from naïve cells to Th17 cells in 8-weeks-old mice (Fig. 4a). We found that TGF-β was significantly increased in the brains of the CPMS group compared to the CTL group (Hip, *p* = 0.0005; Amy, *p* = 0.0002; PFC; *p* = 0.0464; Fig. 4b and Additional file 1: Fig. S6A). Phosphorylated STAT3 (pSTAT3) in the same regions was also found to be significantly increased in the CPMS group (Hip, *p* = 0.0207; Amy, *p* = 0.0369; PFC, *p* = 0.0261; Fig. 4c and Additional file 1: Fig. S6B).

Recently, it has been suggested that prolonged production of IL-17 by Th17 cells is dependent on the actions of the transcription factor RORγt [26]. So, we examined the RORγt levels in the brains of CPMS group mice and found a significant increase in RORγt in the Amy (*p* = 0.0282) and PFC (*p* = 0.0057) (Fig. 4d and Additional file 1: Fig. S6C). The average protein expression level of RORγt also increased in Hip (*p* = 0.0919), but was not significant (Fig. 4d and Additional file 1: Fig. S6C).

To identify a population of Th17 cells in the whole brains of 8-week-old mice, we performed flow cytometry (Additional file 1: Fig. S7). As shown in Fig. 4e, f, the population of CD4^+^IL-17^+^ cell-targeted Th17 cells was increased about 2.4 times in the brain of 8-week-old CPMS (*p* = 0.0059) compared with CTL mice, so IL-17 would have been increased by the increase in the Th17 cell population.

### Anti-IL-17 treatment ameliorated anxiety- and depression-like behaviors in CPMS mice

**Fig. 5:**
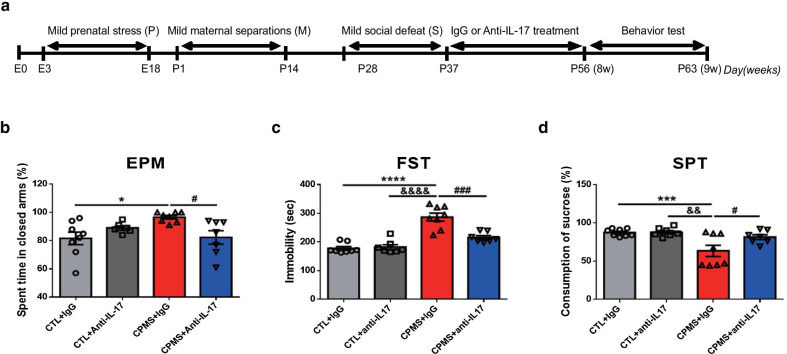
Anti-IL-17 reduced anxiety- and depression-like behavior in CPMS mice. Mice were injected with an anti-IL-17 or IgG (total 5 times for 2 weeks) after cumulative mild stress. **a** Experimental scheme included behavior tests in the CPMS mice model after anti-IL-17 treatment. After treatment, 8-week-old mice were subjected to **b** EPM (CTL + IgG (n = 8); 81.63 ± 4.468, CTL + Anti-IL-17 (n = 6); 89.00 ± 1.528, CPMS + IgG (n = 8); 96.63 ± 1.253, CPMS + Anti-IL-17 (n = 7) 82.29 ± 4.799), **c** FST (CTL + IgG; 177.8 ± 6.256, CTL + Anti-IL-17; 182.4 ± 8.552, CPMS + IgG; 287.3 ± 14.00, CPMS + Anti-IL-17, 215.7 ± 6.707), and **d** SPT (CTL + IgG; 87.38 ± 1.721, CTL + Anti-IL-17; 87.86 ± 2.198, CPMS + IgG; 63.50 ± 7.273, CPMS + Anti-IL-17; 81.43 ± 3.176). Data are expressed as mean ± SEM. *p < 0.05, ***p < 0.001, ****p < 0.0001 vs. CTL + IgG; ^&&^*p* < 0.01, ^&&&&^*p* < 0.0001 vs. CTL + anti-IL-17; ^#^*p* < 0.05, ^###^*p* < 0.001 vs.; CPMS + IgG

To prove that IL-17 is involved in the anxiety- and depression-like behavior of CPMS mice, we suppressed the action of IL-17 through anti-IL-17/IL-17A antibody treatment. After anti-IL-17 or immunoglobulin G treatment, 8-week-old mice were evaluated for anxiety and depression-like behavior (Fig. 5a). In the EPM test, the CPMS + IgG group (0.0242 vs CTL + IgG) exhibited significantly high levels of anxiety compared with the CTL or anti-IL-17 treatment groups, and the CPMS + anti-IL-17 group recovered similarly to the CTL group (F_(3,25)_ = 4.257, *p* = 0.0147, Fig. 5b). Also, immobility time was significantly increased in the CPMS + IgG group (*p* < 0.0001 vs CTL + IgG) in the FST, while anti-IL-17 significantly reduced immobility time of CPMS mice, alleviating despair in CPMS mice (F_(3,26)_ = 28.78, *p* < 0.0001, Fig. 5c). Furthermore, in the SPT, sucrose consumption of the CPMS + IgG group (*p* = 0.0030 vs CTL + IgG) was decreased compared with the CTL group. However, anti-IL-17 treatment significantly increased sucrose consumption in CPMS mice similar to the CTL group (F_(3,26)_ = 7.058, *p* = 0.0013, Fig. 5d), ameliorating anhedonia in the CPMS group.

### Anti-IL-17 treatment ameliorated upregulated inflammatory cytokines IL-17, as well as microglial activation in CPMS mice

**Fig. 6:**
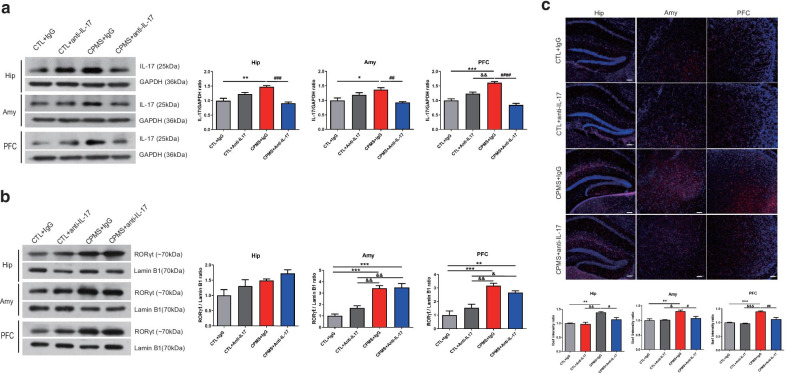
Anti-IL-17 reduced increased inflammatory cytokines and microglia activation in young adult CPMS mice. Brain tissues were collected from mice after treatment with an anti-IL-17 or IgG (total 5 times for 2 weeks) in CPMS mice. Western blot analysis was performed using the cytosol isolated from the hippocampus (Hip), amygdala (Amy), and prefrontal cortex (PFC). **a** RORγt of Hip (CTL + Anti-IL-17; 1.307 ± 0.2092, CPMS + IgG; 1.492 ± 0.04870, CPMS + Anti-IL-17; 1.729 ± 0.1189 vs CTL + IgG), Amy (CTL + Anti-IL-17; 1.681 ± 0.8243, CPMS + IgG; 3.421 ± 0.2366, CPMS + Anti-IL-17; 3.499 ± 0.3410 vs CTL + IgG) and PFC (CTL + Anti-IL-17; 1.527 ± 0.2602, CPMS + IgG; 3.173 ± 0.1740, CPMS + Anti-IL-17; 2.656 ± 0.1406 vs CTL + IgG) was normalized by Lamin B1, and **b** IL-17 of Hip (CTL + Anti-IL-17; 1.228 ± 0.05834, CPMS + IgG; 1.480 ± 0.03881, CPMS + Anti-IL-17; 0.9077 ± 0.04316 vs CTL + IgG), Amy (CTL + Anti-IL-17; 1.191 ± 0.07569, CPMS + IgG; 1.373 ± 0.06446, CPMS + Anti-IL-17; 0.9261 ± 0.02749 vs CTL + IgG) and PFC (CTL + Anti-IL-17; 1.243 ± 0.04680, CPMS + IgG; 1.614 ± 0.03935, CPMS + Anti-IL-17; 0.8410 ± 0.05664 vs CTL + IgG) was normalized by GAPDH. **c** Immunofluorescence staining for Iba1(red) was performed in the 9-week-old mice (n = 3–5 per group). Scale bar 100 μm. Microglia intensity was quantified in Hip (CTL + Anti-IL-17; 0.9702 ± 0.06205, CPMS + IgG; 1.385 ± 0.03117, CPMS + Anti-IL-17; 1.136 ± 0.06541 vs CTL + IgG) ± Amy (CTL + Anti-IL-17; 1.017 ± 0.006536, CPMS + IgG; 1.315 ± 0.03766, CPMS + Anti-IL-17; 1.074 ± 0.9068 vs CTL + IgG), PFC (CTL + Anti-IL-17; 0.9675 ± 0.01086, CPMS + IgG; 1.403 ± 0.02017, CPMS + Anti-IL-17; 1.121 ± 0.06376 vs CTL + IgG). All data are presented as mean ± SEM of three or more independent experiments. *p < 0.05, **p < 0.01, ***p < 0.001 vs CTL + IgG, ^&^*p* < 0.05, ^&&^*p* < 0.01, ^&&&^*p* < 0.001 vs CTL + Anti-IL-17, ^#^*p* < 0.05, ^##^*p* < 0.01, ^###^*p* < 0.001, ^####^*p* < 0.001 vs CPMS + IgG

After the behavior test in CPMS mice treated by anti-IL-17 or immunoglobulin G treatment, we confirmed changes in RORγt and IL-17 levels in the brains of the mice via western blot analysis. The level of RORγt was no difference by anti-IL-17 treatment in the brain compared with CPMS + IgG mice (Fig. 6a). However, Anti-IL-17 significantly reduced the levels of IL-17 in Hip (F_(3,8)_ = 19.80, *p* = 0.0005), Amy (F_(3,8)_ = 8.971, *p* = 0.0061) and PFC (F_(3,8)_ = 43.42, *p* < 0.0001) compared with CPMS + IgG mice (Fig. 6b).

Next, we evaluated for the intensity of microglia in the Hip (F_(3,11)_ = 11.69, *p* = 0.0010), Amy (F_(3,11)_ = 8.157, *p* = 0.0039), and PFC (F_(3,11)_ = 17.85, *p* = 0.0002) in the CPMS group compared to the CTL group (Fig. 6c). Espceically, Iba1 intensity was significantly decreased in the CPMS + anti-IL-17 group compared with the CPMS + IgG group (Hip, *p* = 0.0279; Amy, *p* = 0.0230; PFC, *p* = 0.0038; Fig. 6b).

## Discussion

An adverse environment in early life has a significant influence on behavioral or psychological changes in young adulthood [27, 28]. Negative inputs, such as acute or chronic stress during development, may lead to changes in gene expression or stress-responsive systems, and ultimately to psychopathology with an abnormal inflammatory response involving complex processes [29, 30]. One of the important characteristics of MDD compared the other disease related to stress such as post-traumatic stress disorder (PTSD) is the accumulation of stress memories in the past [31]. Also, multiple stressors accumulating and combining to lead to more severe outcomes such as abnormal inflammation in the developmental literature [32, 33]. Although there are many mice models of depression, the periods or stressors were limited. So we generated a CPMS mouse model for depression in young adulthood by exposing cumulative three kinds of mild stress (P, M, and S) in early life using a modified protocol that complements the previous mouse model based on stress [34]. During the periods from embryo to adulthood, neurogenesis, synaptogenesis, and myelination as well as circuit formation of diverse functional lobes occur actively [35, 36]. Thus, cumulative mild stress throughout the growth process can alter emotional regulation, metabolism, and immune systems [37]. Assessment of rodent depression models should include behavioral tests to assess clinically relevant depressive behavioral symptoms [38]. CPMS mice also exhibited long-lasting anxiety- or depression-like behavior, which are the representative behaviors such as despair or anhedonia in human MDD patients. However, thorough the result of normal movement in CPMS mice, we confirmed that the stress applied to the mouse model generation was not severe or excessive (Additional file 1: Fig. S2). Furthermore, we compared the CPMS mice model with the other models exposing single or double mild stressors. Interestingly, there was no significant in despair of the S group under mild social defeat stress, but the double combination mild stress groups such as PS or MS, applied during the prenatal or infant period, showed significantly elevated despair. However, only the CPMS group showed significantly increased anhedonia in SPT. These results demonstrated that the degree of mild stress accumulation is associated with chronic depressive symptoms. To approve as depression models, pharmacological validity, which requires the reversal of depressive symptoms by available anti-depressants, need to be met [39]. Therefore, we also demonstrated that anxiety- and depression-like behaviors of CPMS mice were recovered by venlafaxine treatment, which inhibits the 5-HT/noradrenalin synaptosomal uptake [40]. Venlafaxine also has anti-inflammatory property about abnormal inflammatory responses related to microglia as one of the risk factors causing depressive symptoms [41–43]. So, we confirmed that Velafaxine reduced microglial activation (Additional file 1: Fig. S2C) and it was considered that Venlafaxine may have rescued depressive behavior in CPMS animals by supressing the upregulated inflammatory response. These results support that the cumulative mild stress may mimic the growth circumstances of patients with MDD better than other stressors and the CPMS model was appropriate to use in MDD with young adulthood.

Since behavioral tests such as FST can affect mouse models, we newly created the CPMS mice to measure stress-related molecular changes. It has been known that chronic stress causes loss of body weight and increases corticosterone levels in serum [44]. Consistent with these results, CPMS mice had lower body weight and higher serum corticosterone levels compared to CTL mice (Additional file 1: Fig. S3). Indeed, early life stress may impact the inflammatory reactions in adults by enhancing the transcription of nuclear factor kappa B (NF-κB) by increasing pro-inflammatory cytokines [33]. In normal conditions, stress commonly triggers the hypothalamic–pituitary–adrenal (HPA) axis through the corticosterone releasing hormone (CRH), which usually suppresses immune response via the release of glucocorticoids (GC) from adrenals by inhibiting NF-κB [45, 46]. Prolonged hypersensitivity to stress with exaggerated circulatory GC could destroy the negative feedback loop of the HPA axis, triggering behaviors such as anxiety and depression [47].

In our study, we found that cumulative mild stress consistently induces excessive cytokines in the brains and peripheral blood of young adult CPMS mice. These results are consistent with the results of a meta-analysis showing that patients who experienced early life adversity had a marked increase in baseline peripheral levels of IL-6, TNF-α, and CRP compared to those who did not [48]. Another previous study showed that the consequences of early life stress-induced infection altered cytokine production, such as IL-6, TNF-α, or IL-1β, and changed behaviors in later life [49]. Our results were similar to the previous clinical report that both levels of IL-18 and IL-18/IL-18BP ratios were significantly higher in patients with early-onset psychosis (EOP) aged 12–18 years than in age-matched healthy controls [50].

Recently, some clinical or preclinical studies reported that IL-17 and Th 17 cells were associated with MDD [13–15, 51]. Interestingly, the CPMS mice had elevated IL-17 levels in the Hip, Amy, and PFC regions of the brain as well as activated microglia. In previous reports, IL-17 promoted the production of several cytokines, chemokines, and nitric oxide, which damaged the brain both directly and indirectly via the disruption of the blood–brain barrier [52, 53]. IL-17 also induced structural remodeling of microglia and enhanced the release of pro-inflammatory cytokines from microglia [54]. Through these results, we hypothesized that increased IL-17 caused by cumulative mild stress in early life plays an important mediator in inducing and sustaining anxiety- and depression-like behavior in young adulthood. To prove our hypothesis, we compared the depressive symptoms and the level of IL-17 in the CPMS group with a single or double combinations group of mild stress including mild social defeat stress. As a result, IL-17 levels in the brain of CPMS mice were higher than single (S) or double stressor groups (PS, MS), and the degree of depressive symptoms was correlated with increased levels of IL-17 in the brain (Additional file 1: Fig. S8). Additionally, we found an increase in the differentiation factors and population of Th17 cells, which produce IL-17, in the brains of CPMS mice. Finally, we identified the association between IL-17 and depressive symptoms in CPMS mice, via anti-IL-17 treatment to inhibit potent IL-17. These results suggested that cumulative mild stress in early life induced excessive IL-17, which drove from Th 17 cells, and it may promote a persistent inflammatory response, which can occur brain damage. These abnormal responses of inflammation as a result of cumulative mild stress during brain development may occur depressive symptoms in young adulthood.

The limitations of this study include only male mouse results from inflammation-related experimental data that can be comparable to behavioral results. If additional experiments are carried out with the female CPMS group, we can improve our understanding of gender differences of MDD pathophysiology that occurred by cumulative stresses in early life. In addition, more detailed changes in the structure and connectivity should be investigated in the brains of the CPMS mouse model in future studies.

**Fig. 7:**
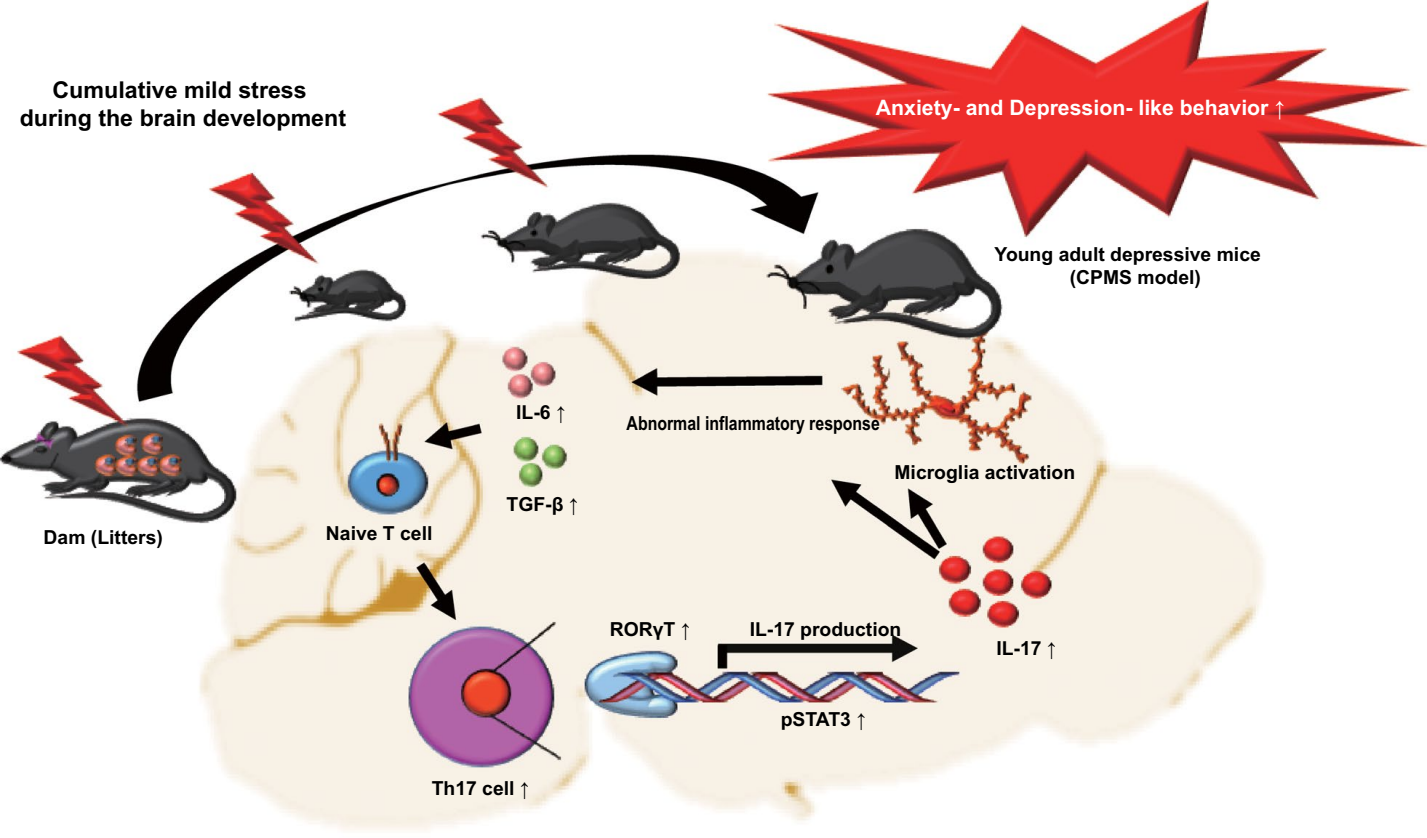
Summary picture. Cumulative mild stress during brain development (from fetus to adolescence) induced long-lasting depressive symptoms in young adulthood. Naïve T cell was differentiated to Th17 cells under the increase of differentiation factors such as IL-6 and TGF-β with the hyperactive form of STAT3. According to the increase of Th17 cell population, the high level of IL-17 was produced and promoted the activation of microglia. As a result, the abnormal inflammatory response induced by IL-17 affects more long-lasting anxiety- and depression-like behavior in young adulthood

In summary, through the generation of a mouse model for young adult depression, we identified that cumulative mild stress during brain development incurred long-lasting anxiety- and depression-like behavior with the upregulated inflammatory response in young adulthood (Fig. 7). Furthermore, we demonstrated that IL-17 may be one of the factors associated with long-lasting behavioral symptoms. We anticipated that our finding may be a diagnosis or therapeutic target for depression in young adulthood.

## Materials and methods

### Experimental animals

Male and female C57BL/6N mice were purchased from Dae Han Bio-Link Co., Ltd (Eumseong, Korea) and were bred according to the schedule after undergoing an adaptation period. The mice were housed under a 12:12 h light–dark cycle with 22 ± 2 ℃ temperature and 40–60% humidity and were given free access to food and water except under stress conditions. All animal procedures were approved by the Institutional Animal Care and Use Committee of the Lee Gil Ya Cancer and Diabetes Institute, Gachon University (IACUC No. LCDI-2016-0100).

### Cumulative mild stress procedure

The mice underwent cumulative mild stress during the critical periods of brain development from fetuses to adolescence (Additional file 1: Fig. S1). The cumulative mild stress included mild prenatal stress (P), mild maternal separation (M), and mild social defeat (S). In short, pregnant mice were exposed to unpredictable mild stress (Table 1) according to a modified protocol [55]. After litters were born, the postnatal day 2 (P2) offspring were separated from their mother for 3 h per day for 14 days by placing them in an individual space maintained under normal conditions. The mild maternal separation stress protocol was also modified from a previously reported protocol [56]. When the mice were 4 weeks old, they were exposed to mild social defeat stress for 10 days according to the modified schedules of the previous paradigm [57, 58] shown in Additional file 1: Fig. S1.

**Table 1:** Chronic unpredictable mild stress schedule

	Monday	Tuesday	Wednesday	Thursday	Friday	Saturday	Sunday
9 am							
10 am	Restraint stress (1 h)	Forced swim (5 min)	Restraint stress (1 h)	Cage tilt 30° (6 h)	Food restriction (6 h)		
11 am	10 am–11 am		10 am–11 am	10 am–4 pm	10 am–4 pm		
12 am							
1 pm							
2 pm							
3 pm							
4 pm							
5 pm	Paired housing (overnight)	Continuous lightning (overnight)	Foreign object in cage (overnight)	Soiled cage (overnight)	Paired housing (overnight)	Continuous lightning (overnight)	Cage tilt 30° (overnight)

### Drug administration

Venlafaxine (Sigma Aldrich, St. Louis, MO, USA), a serotonin-norepinephrine reuptake inhibitor (SNRI) anti-depressant, was dissolved in normal saline [40]. Anti-IL-17 (Bioxcell, Lebanon, NH, USA) and IgG (Bioxcell) was dissolved in purified dilution buffer [59]. The mice were treated with venlafaxine (10 mg/kg, once a day for 3 weeks, i.p) or Anti-IL-17 (100 μg/mouse, once every 3 days for 2 weeks, i.p). The same volume of saline or IgG was administrated to mice in the vehicle group, respectively.

### Elevated plus maze test

The elevated plus maze (EPM) consisted of two closed arms (20 cm × 5 cm × 50 cm) and two open arms (20 cm × 5 cm × 50 cm). Mice were placed in the center zone and allowed to freely explore the maze for 5 min. The duration time in all arms was recorded using a video tracking system.

### Forced swimming test

The forced swimming test (FST) was conducted on two consecutive days as previously described with minor modifications [60, 61]. On the first day, the mice were placed in individual cylinders (12 cm diameter and 31 cm height) filled with water (23 ± 1 °C) for 8 min for training. Thereafter, the mice were dried and returned to their home cage. After 24 h in the same condition, total immobility time was recorded using a video camera and was manually measured during the final 6 min.

### Sucrose preference test

One week before the sucrose preference test (SPT), mice were adapted to provided two bottles of water for 5 days. The mice fasted for 16 h before the SPT and were free to access two bottles of water during the fasting period. On the day of the test, the mice were transferred to a single cage for volume determination [62]. Two bottles, one containing 2% sucrose and the other containing water, were measured before testing and the consumed volume was checked after 2 h. Anhedonia was calculated as a ratio of the weight of sucrose solution intake to the weight of total solution consumption according to the following formula:

$${\text{Anhedonia }} = \frac{Sucrose \;intake}{{Sucrose\; intake + Water\; intake }} \left( \% \right).$$

### Collection of blood and brain tissue from mice

The mice were anesthetized with a mixture of Zoletil (16.7 mg/kg) and Rompun (15.5 mg/kg). The blood was collected from the abdominal inferior vena cava and was centrifuged at 3000 rpm for 10 min at 4 °C. The supernatant was transferred into a new tube and protease inhibitor was added (Calbiochem, San Diego, CA, USA). For immunofluorescence, the brain was perfused with saline containing heparin and fixed in 4% paraformaldehyde at 4 °C. After 24 h, the brain was dehydrated in 30% sucrose solution for 72 h at 4 °C. For western blot analysis, the hippocampus (Hip), amygdala (Amy), and prefrontal cortex (PFC) were dissected from non-perfused brain, snap-frozen in liquid nitrogen, and stored at − 80 °C for later use.

### Immunofluorescence

Frozen blocks of brain tissues were coronally cut into 25 μm-thick slices using a cryostat (Cryotome, Thermo Electron Corporation, Waltham, MA, USA), and these were stored in cryoprotectant solution at 4 °C. After washing in PBST (normal saline containing 0.2% Triton X-100), the brain slices were blocked in PBST containing 0.5% BSA and 3% normal goat serum and were incubated with anti-Iba1 antibody (Table 2). The next day, the slices were incubated with an Alexa Fluor 555 conjugated goat anti-rabbit IgG antibody (Invitrogen, CA, USA). The brain slices were then washed and mounted onto slides using Antifade Mounting Medium with DAPI (Vector Laboratories, Burlingame, CA, USA). Specimens were examined under a Nikon TS2-S-SM microscope (Nikon Microscopy, Tokyo, Japan) equipped with a Nikon DS-Qi2 camera. Serial images of 100 × magnification (Hip, Amy, and PFC) were captured from 4 sections per animal. Iba1 stained brain slides of each group were compared and analyzed by region of interest (ROI) intensity ratio (%) using NIS-Elements software (4.40.00 64-bit). Once the ROIs were defined, the red channel showing Alexa Fluro 555 was used to measure both the fluorescence intensity and percentage area of a red signal within each ROI per section (n = 3 per animal).

**Table 2:** Antibody list

Target	Species	Dilution	Company (catalog)
For Western blot analysis			
Anti-CD86	Mouse	1:200	Santa cruz (sc-19617)
Anti-GAPDH	Rabbit	1:5000	Bioworld (A531)
Anti-IL-1β	Rabbit	1:2000	Abcam (ab9722)
Anti-IL-6	Rabbit	1:500	MyBioSource (MBS2002878)
Anti-IL-17	Mouse	1:200	Santa cruz (sc-374218)
Anti-IL-18	Rabbit	1:500	Abnova (PAB16177)
Anti-Lamin B1	Rabbit	1:1000	Abcam (ab65986)
Anti-Phospho STAT3 (Tyr705)	Rabbit	1:1000	Cell signal (9131)
Anti-RORγT	Goat	1:1000	Thermo (14-6981-82)
Anti-TGF-β	Rabbit	1:1000	Cell signal (3711S)
Anti-TNF-α	Mouse	1:500	Santa cruz (sc-52746)
Anti-Total STAT3	Rabbit	1:1000	Cell signal (4904)
Anti-mouse IgG HRP		1:5000–1:10,000	Biorad (170-6516)
Anti-rabbit IgG HRP		1:5000–1:10,000	Biorad (170-6515)
Anti-rat IgG HRP		1:5000–1:10,000	Santa cruz (sc-2006)
For immunofluorescence staining			
Anti-Iba1	Rabbit	1:500	Novus (NBP2-19019)
Alexa Fluor 555 Goat anti rabbit IgG		1:500–1:1000	Invitrogen (A-21429)

### Western blot analysis

Brain tissues were lysed with Syn-PER Synaptic Protein Extraction Reagent (Thermo Scientific, Waltham, MA, USA) containing protease inhibitor and the cocktail of phosphatase inhibitors (Sigma Aldrich). After quantification using the Bradford assay (Bio-Rad Laboratories, Inc., Hercules, CA, USA), cytosolic samples were loaded onto an 8–15% SDS-PAGE and then transferred onto a PVDF membrane (Merck, Kenilworth, NJ, USA). The membrane was blocked with 3% BSA or 6% skim milk at RT for 1 h, incubated overnight with the appropriate primary antibody (Table 2), and then incubated with the corresponding secondary antibody at RT for 1 h after washing. Protein bands were detected using Immobilon Western Chemiluminescent HRP Substrate (Millipore, Burlington, MA, USA) and BLUE X-ray film (AGFA, Mortsel, Belgium). The density of the bands was quantified using ImageJ software v1.4.3.67 (NIH, USA).

### Flow cytometry

Following the perfusion, isolated brains were dissociated and chopped into small pieces, and then they were filtered through a 70 μm cell strainer and centrifuged at 1300 rpm for 5 min. The cell suspension was mixed with 30% Percoll/HBSS medium, overlaid on 70% Percoll/HBSS medium and then centrifuged at 2000 rpm for 30 min without using the brake. After the cells between the 30/70% Percoll gradients were recovered, they were washed once. The cells in media were stimulated with activation Cocktail (BioLegend, San Diego, CA, USA) for 4 h at the recommended concentrations. The cells were blocked with a purified anti-mouse CD16/32 (BioLegend) antibody and stained extracellularly with a PerCP/Cyanine5.5-conjugated anti-mouse CD45 antibody (BioLegend) and FITC-conjugated anti-mouse CD4 antibody (BioLegend). To intracellularly stain the cells, they were fixed and permeabilized using a Fixation/Permeabilization Solution Kit (BD Bioscience, San Jose, CA, USA) and stained with an Alexa Fluor 647-conjugated anti-mouse IL-17A antibody (BioLegend). Data were acquired using an LSRII flow cytometer (BD Biosciences) and analyzed with FlowJo software version 8.7 (FlowJo, LCC, Ashland, OR, USA).

### Statistical analysis

All data are presented as mean ± standard error of the mean (SEM). Statistical analysis was performed using GraphPad Prism 8.4.2 (679) software (GraphPad Software Inc., San Diego, CA, USA). Two group comparison (e.g. western blot, immunofluorescence intensity, FACS, and ELISA) was analyzed by unpaired t test and multiple group comparisons were analyzed by One-way (e.g. behavior test, western blot) or Two-way ANOVA (e.g. measurement of body weight) followed by Bonferroni’s post hoc test. *p* < 0.05 was considered statistically significant for all analyses.

## Supplementary Information

**Additional file 1**. Supplementary information.

## Data Availability

All data generated or analyzed during this study are included in this published article [and its supplementary information].

## References

[1] Katon WJ (2003). Clinical and health services relationships between major depression, depressive symptoms, and general medical illness. Biol Psychiatry.

[2] Lu Y, Tang C, Liow CS, Ng WW, Ho CS, Ho RC (2014). A regressional analysis of maladaptive rumination, illness perception and negative emotional outcomes in Asian patients suffering from depressive disorder. Asian J Psychiatr.

[3] Sherdell L, Waugh CE, Gotlib IH (2012). Anticipatory pleasure predicts motivation for reward in major depression. J Abnorm Psychol.

[4] Kessler RC, Ustun TB (2004). The World Mental Health (WMH) Survey Initiative Version of the World Health Organization (WHO) Composite International Diagnostic Interview (CIDI). Int J Methods Psychiatr Res.

[5] Horowitz JL, Garber J (2006). The prevention of depressive symptoms in children and adolescents: a meta-analytic review. J Consult Clin Psychol.

[6] Gayman MD, Lloyd DA, Ueno K (2011). The history and timing of depression onset as predictors of young-adult self-esteem. J Res Adolesc.

[7] Zisook S, Lesser I, Stewart JW, Wisniewski SR, Balasubramani GK, Fava M (2007). Effect of age at onset on the course of major depressive disorder. Am J Psychiatry.

[8] Beautrais AL (2002). Gender issues in youth suicidal behaviour. Emerg Med (Fremantle).

[9] Mahmoud JS, Staten R, Hall LA, Lennie TA (2012). The relationship among young adult college students' depression, anxiety, stress, demographics, life satisfaction, and coping styles. Issues Ment Health Nurs.

[10] Syed SA, Nemeroff CB (2017). Early life stress, mood, and anxiety disorders. Chronic Stress (Thousand Oaks)..

[11] Goodwill HL, Manzano-Nieves G, Gallo M, Lee HI, Oyerinde E, Serre T (2019). Early life stress leads to sex differences in development of depressive-like outcomes in a mouse model. Neuropsychopharmacology.

[12] Chen Y, Baram TZ (2016). Toward understanding how early-life stress reprograms cognitive and emotional brain networks. Neuropsychopharmacology.

[13] Nadeem A, Ahmad SF, Al-Harbi NO, Fardan AS, El-Sherbeeny AM, Ibrahim KE (2017). IL-17A causes depression-like symptoms via NFkappaB and p38MAPK signaling pathways in mice: implications for psoriasis associated depression. Cytokine.

[14] Davami MH, Baharlou R, Ahmadi Vasmehjani A, Ghanizadeh A, Keshtkar M, Dezhkam I (2016). Elevated IL-17 and TGF-beta serum levels: a positive correlation between T-helper 17 cell-related pro-inflammatory responses with major depressive disorder. Basic Clin Neurosci.

[15] Chen Y, Jiang T, Chen P, Ouyang J, Xu G, Zeng Z (2011). Emerging tendency towards autoimmune process in major depressive patients: a novel insight from Th17 cells. Psychiatry Res.

[16] Beurel E, Harrington LE, Jope RS (2013). Inflammatory T helper 17 cells promote depression-like behavior in mice. Biol Psychiatry.

[17] Waisman A, Hauptmann J, Regen T (2015). The role of IL-17 in CNS diseases. Acta Neuropathol.

[18] Korn T, Bettelli E, Gao W, Awasthi A, Jager A, Strom TB (2007). IL-21 initiates an alternative pathway to induce proinflammatory T(H)17 cells. Nature.

[19] Ivanov II, McKenzie BS, Zhou L, Tadokoro CE, Lepelley A, Lafaille JJ (2006). The orphan nuclear receptor RORgammat directs the differentiation program of proinflammatory IL-17+ T helper cells. Cell.

[20] Yang XO, Pappu BP, Nurieva R, Akimzhanov A, Kang HS, Chung Y (2008). T helper 17 lineage differentiation is programmed by orphan nuclear receptors ROR alpha and ROR gamma. Immunity.

[21] Das J, Ren G, Zhang L, Roberts AI, Zhao X, Bothwell AL (2009). Transforming growth factor beta is dispensable for the molecular orchestration of Th17 cell differentiation. J Exp Med.

[22] Zhou L, Ivanov II, Spolski R, Min R, Shenderov K, Egawa T (2007). IL-6 programs T(H)-17 cell differentiation by promoting sequential engagement of the IL-21 and IL-23 pathways. Nat Immunol.

[23] Wienke J, Janssen W, Scholman R, Spits H, van Gijn M, Boes M (2015). A novel human STAT3 mutation presents with autoimmunity involving Th17 hyperactivation. Oncotarget.

[24] Malykhin NV, Carter R, Hegadoren KM, Seres P, Coupland NJ (2012). Fronto-limbic volumetric changes in major depressive disorder. J Affect Disord.

[25] Suzuki H, Botteron KN, Luby JL, Belden AC, Gaffrey MS, Babb CM (2013). Structural-functional correlations between hippocampal volume and cortico-limbic emotional responses in depressed children. Cogn Affect Behav Neurosci.

[26] Guendisch U, Weiss J, Ecoeur F, Riker JC, Kaupmann K, Kallen J (2017). Pharmacological inhibition of RORgammat suppresses the Th17 pathway and alleviates arthritis in vivo. PLoS ONE.

[27] Bolton JL, Molet J, Ivy A, Baram TZ (2017). New insights into early-life stress and behavioral outcomes. Curr Opin Behav Sci.

[28] Liu RT (2017). Childhood adversities and depression in adulthood: current findings and future directions. Clin Psychol (New York).

[29] Masten AS, Cicchetti D (2010). Developmental cascades. Dev Psychopathol.

[30] Klein DN, Arnow BA, Barkin JL, Dowling F, Kocsis JH, Leon AC (2009). Early adversity in chronic depression: clinical correlates and response to pharmacotherapy. Depress Anxiety.

[31] Richter-Levin G, Xu L (2018). How could stress lead to major depressive disorder?. IBRO Rep.

[32] Suliman S, Mkabile SG, Fincham DS, Ahmed R, Stein DJ, Seedat S (2009). Cumulative effect of multiple trauma on symptoms of posttraumatic stress disorder, anxiety, and depression in adolescents. Compr Psychiatry.

[33] Fagundes CP, Way B (2014). Early-life stress and adult inflammation. Curr Dir Psychol Sci.

[34] Planchez B, Surget A, Belzung C (2019). Animal models of major depression: drawbacks and challenges. J Neural Transm (Vienna).

[35] Tau GZ, Peterson BS (2010). Normal development of brain circuits. Neuropsychopharmacology.

[36] Stagni F, Giacomini A, Guidi S, Ciani E, Bartesaghi R (2015). Timing of therapies for Down syndrome: the sooner, the better. Front Behav Neurosci.

[37] Teicher MH, Samson JA, Anderson CM, Ohashi K (2016). The effects of childhood maltreatment on brain structure, function and connectivity. Nat Rev Neurosci.

[38] Soderlund J, Lindskog M (2018). Relevance of rodent models of depression in clinical practice: can we overcome the obstacles in translational neuropsychiatry?. Int J Neuropsychopharmacol.

[39] Krishnan V, Nestler EJ (2011). Animal models of depression: molecular perspectives. Curr Top Behav Neurosci.

[40] Wang CH, Gu JY, Zhang XL, Dong J, Yang J, Zhang YL (2016). Venlafaxine ameliorates the depression-like behaviors and hippocampal S100B expression in a rat depression model. Behav Brain Funct.

[41] Kamel KM, Gad AM, Mansour SM, Safar MM, Fawzy HM (2019). Venlafaxine alleviates complete Freund's adjuvant-induced arthritis in rats: modulation of STAT-3/IL-17/RANKL axis. Life Sci.

[42] Vollmar P, Haghikia A, Dermietzel R, Faustmann PM (2008). Venlafaxine exhibits an anti-inflammatory effect in an inflammatory co-culture model. Int J Neuropsychopharmacol.

[43] Zhang YB, Bi XY, Adebiyi O, Wang JH, Mooshekhian A, Cohen A (2019). Venlafaxine improves the cognitive impairment and depression-like behaviors in a cuprizone mouse model by alleviating demyelination and neuroinflammation in the brain. Front Pharmacol..

[44] Jeong JY, Lee DH, Kang SS (2013). Effects of chronic restraint stress on body weight, food intake, and hypothalamic gene expressions in mice. Endocrinol Metab (Seoul).

[45] Danese A, Pariante CM, Caspi A, Taylor A, Poulton R (2007). Childhood maltreatment predicts adult inflammation in a life-course study. Proc Natl Acad Sci USA.

[46] Miller GE, Chen E, Sze J, Marin T, Arevalo JM, Doll R (2008). A functional genomic fingerprint of chronic stress in humans: blunted glucocorticoid and increased NF-kappaB signaling. Biol Psychiatry.

[47] Maniam J, Antoniadis C, Morris MJ (2014). Early-life stress, HPA axis adaptation, and mechanisms contributing to later health outcomes. Front Endocrinol (Lausanne).

[48] Baumeister D, Akhtar R, Ciufolini S, Pariante CM, Mondelli V (2016). Childhood trauma and adulthood inflammation: a meta-analysis of peripheral C-reactive protein, interleukin-6 and tumour necrosis factor-alpha. Mol Psychiatry.

[49] Bilbo SD, Schwarz JM (2009). Early-life programming of later-life brain and behavior: a critical role for the immune system. Front Behav Neurosci.

[50] Wedervang-Resell K, Friis S, Lonning V, Smelror RE, Johannessen C, Reponen EJ (2020). Increased interleukin 18 activity in adolescents with early-onset psychosis is associated with cortisol and depressive symptoms. Psychoneuroendocrinology.

[51] Beurel E, Lowell JA (2018). Th17 cells in depression. Brain Behav Immun.

[52] Cipollini V, Anrather J, Orzi F, Iadecola C (2019). Th17 and cognitive impairment: possible mechanisms of action. Front Neuroanat.

[53] Osborne LM, Brar A, Klein SL (2019). The role of Th17 cells in the pathophysiology of pregnancy and perinatal mood and anxiety disorders. Brain Behav Immun.

[54] Yu A, Duan H, Zhang T, Pan Y, Kou Z, Zhang X (2016). IL-17A promotes microglial activation and neuroinflammation in mouse models of intracerebral haemorrhage. Mol Immunol.

[55] Willner P, Towell A, Sampson D, Sophokleous S, Muscat R (1987). Reduction of sucrose preference by chronic unpredictable mild stress, and its restoration by a tricyclic antidepressant. Psychopharmacology.

[56] Sanders BJ, Anticevic A (2007). Maternal separation enhances neuronal activation and cardiovascular responses to acute stress in borderline hypertensive rats. Behav Brain Res.

[57] Kudryavtseva NN, Bakshtanovskaya IV, Koryakina LA (1991). Social model of depression in mice of C57BL/6J strain. Pharmacol Biochem Behav.

[58] Iniguez SD, Riggs LM, Nieto SJ, Dayrit G, Zamora NN, Shawhan KL (2014). Social defeat stress induces a depression-like phenotype in adolescent male c57BL/6 mice. Stress.

[59] Faraco G, Brea D, Garcia-Bonilla L, Wang G, Racchumi G, Chang H (2018). Dietary salt promotes neurovascular and cognitive dysfunction through a gut-initiated TH17 response. Nat Neurosci.

[60] Porsolt RD, Bertin A, Jalfre M (1977). Behavioral despair in mice: a primary screening test for antidepressants. Arch Int Pharmacodyn Ther.

[61] Petit-Demouliere B, Chenu F, Bourin M (2005). Forced swimming test in mice: a review of antidepressant activity. Psychopharmacology.

[62] Ueno H, Shimada A, Suemitsu S, Murakami S, Kitamura N, Wani K (2019). Anti-depressive-like effect of 2-phenylethanol inhalation in mice. Biomed Pharmacother.

