# Supplementary material for: Interleukin-17 induced by cumulative mild stress promoted depression-like behaviors in young adult mice
Source: Mol Brain. 2021 Jan 13;14:11. doi: 10.1186/s13041-020-00726-x (PMC7805143; doi:10.1186/s13041-020-00726-x)
Supplement: Supplementary file 1 — Additional file 1. Supplementary information. [file 13041_2020_726_MOESM1_ESM.pdf]

Supplementary methods

Open-field test

The open-field box (40 cm x 40 cm x 40 cm) consisted of four black polyvinyl chloride (PVC) walls and a white PVC floor. The mice were allowed to move freely within the box, and their activity was monitored for 5 min using a ceiling-mounted video camera. To confirm normal locomotor activity, the total distance traveled during the first 5 min was calculated.

Y-maze test

The Y-maze apparatus consisted of three white PVC arms (5 cm x 40 cm x 10 cm) positioned at an angle of 120° to one another. The mice were placed in the center of the maze and allowed to explore each arm freely for 8 min. The total number of arm entries and entering sequence was recorded using a recording system. The percentage of spontaneous alternation was calculated according to the following formula:

$$\text{Spontaneous alternation} = \frac{\text{Number of consecutive entries into three arms } (A,B,C)}{Total\ number\ of\ entries - 2}\ (\%)$$

Enzyme-linked immunosorbent assay

The levels of corticosterone, IL-6, IL-17, and TNF-α, in serum, were analyzed using commercial enzyme-linked immunosorbent assay (ELISA) kits according to the manufacturer's instructions (corticosterone: Enzo, Madison Avenue, NY, USA; IL-6: Abbkine, Wuhan, China; IL-17: R&D Systems, Minneapolis, MN, USA, and TNF-α: Abbkine, KET7015;). The protein levels of corticosterone and inflammatory cytokines in serum were assayed in duplicate and measured using a VICTOR X4 Multimode Plate Reader (PerkinElmer, *Waltham, MA, USA*).

Supplementary figures

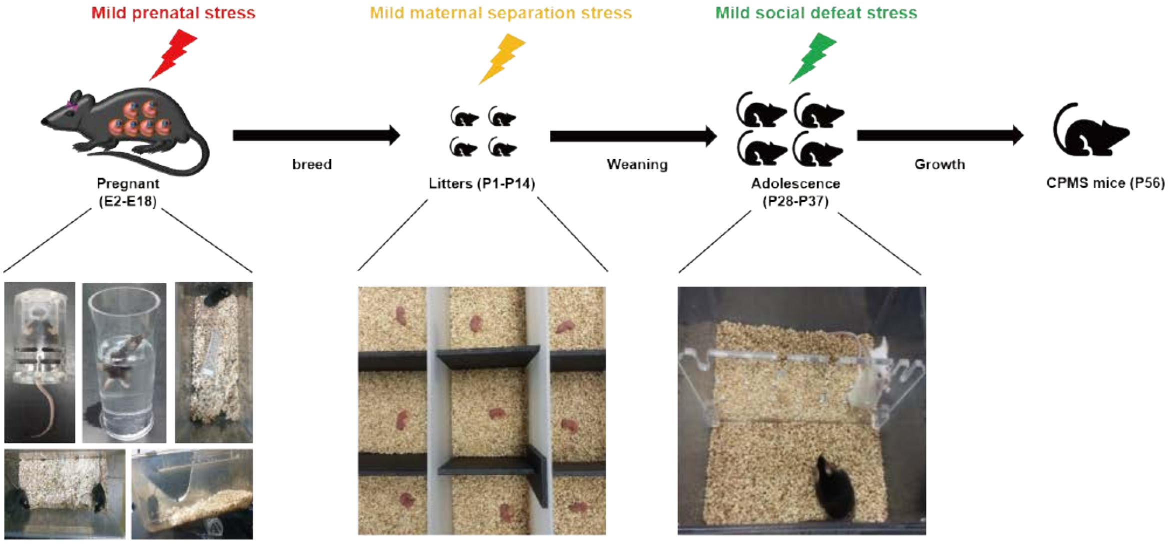

Fig.S1 Representative image for the creation of the CPMS mice model

Following the 1-week habituation, 7- to 8-week-old male and female C57BL/6N mice were mated. Early the next morning, we checked for pregnancy by checking for the presence of a vaginal plug. Each confirmed pregnant mouse was separated into a single cage at timed E2. The fetuses were indirectly exposed to mild prenatal stress through their mother which suffered unpredictable mild stress (Table 1) from E3 to E18. After birth, P2 old litters underwent mild maternal separation for 16 days. Mild maternal separation stress was performed for 3 h per day with normal bedding and temperature (22 ± 2°C) conditions. After weaning, 4-week-old C57BL/6N mice were daily harassed by ICR male mouse for 5 min. Following its defeat, the C57BL/6N mouse was moved to the opposite side of the partition for 3 min, which enabled it to see and smell in the same cage but blocked physical contact. After mild social defeat stress, the C57BL/6N mouse was returned to its home cage. E: embryonic (prenatal) day, P: postnatal day.

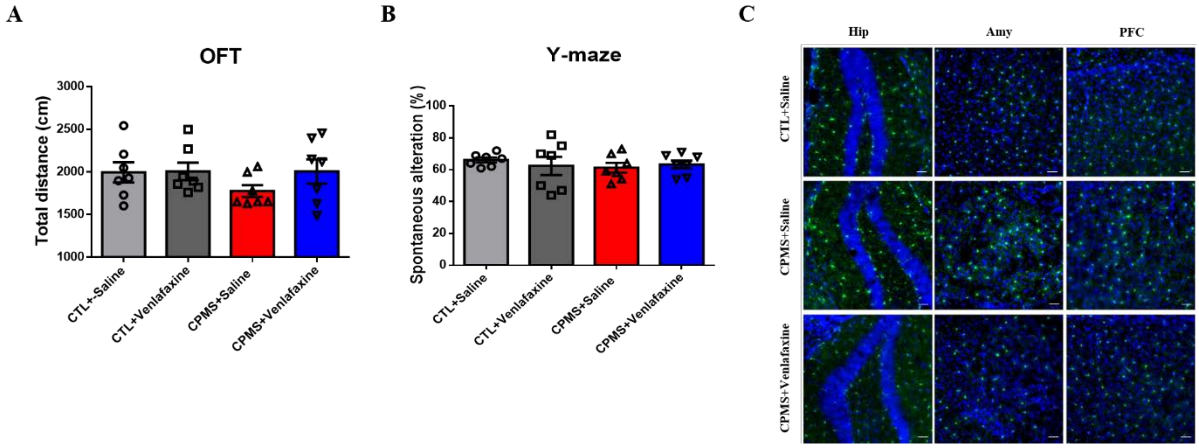

Fig.S2 No significant difference between both movements in OFT and short-term memory in Y-maze test

Following cumulative mild stress and Venlafaxine treatment as with Fig. 1, the mice (n=7 per group) were examined for locomotor activity and short-term memory using the open-field test and Y-maze test. (A) Total distance (CTL-saline; 1994 ± 118.2, CTL-Venlafaxine; 2006 ± 103.1, CPMS-saline; 1774 ± 69.26, CPMS-Venlafaxine; 2005 ± 141.6) and (B) spontaneous alteration (CTL-saline; 66.14 ± 1.503, CTL-Venlafaxine; 62,43 ± 5.719, CPMS-saline; 61,29 ± 3.068, CPMS-Venlafaxine; 6.701±2.553) produced no significant differences among the groups. One-way ANOVA was used to analyze behavioral tests. (C) Immunofluorescence staining for Iba1(green) was performed in the 9-week-old mice. DAPI was counterstained to detect nuclei. scale bar = 100μm.

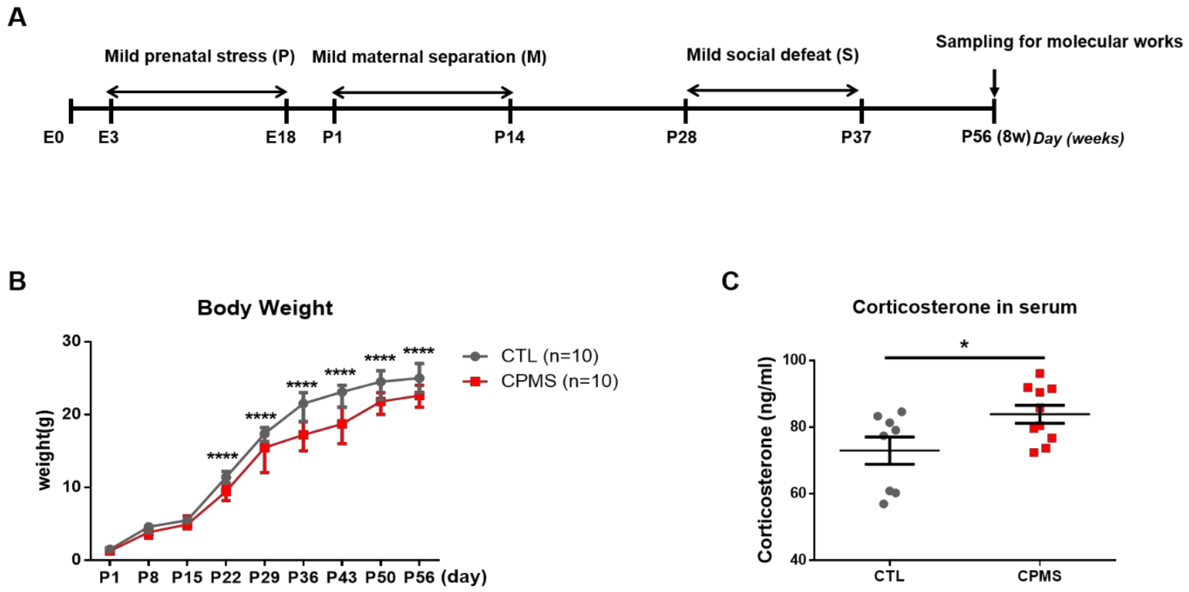

Fig.S3 Measurement of body weight and corticosterone

(A) To browse the changes in the brain and peripheral blood, the CPMS mice were newly generated in the same way. (B) The bodyweight of mice (n=10 per group) was measured from P1 to P56. Two-way ANOVA was used for analysis between the groups. Following cumulative mild stress, serum was isolated from 8-week-old mice. (C) The corticosterone (CTL=8, CPMS=10) was examined using ELISA analysis. An unpaired t-test was used for the comparison between the regions. *$p<0.05$, ****$p<0.0001$ vs. CTL.

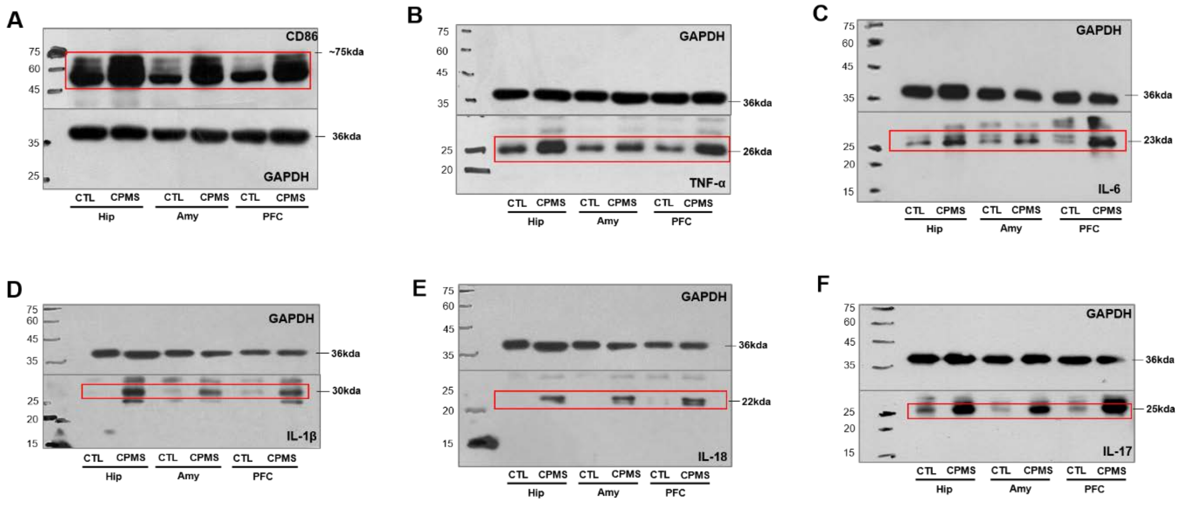

Fig.S4 The full blots of Fig.3

The band in the red box was read for quantification. (A) CD86, (B) TNF-α, (C) IL-6, (D) IL-1β, (E) IL-18 and (F) IL-17 were normalized by GAPDH. An unpaired t-test was used for analysis in each region.

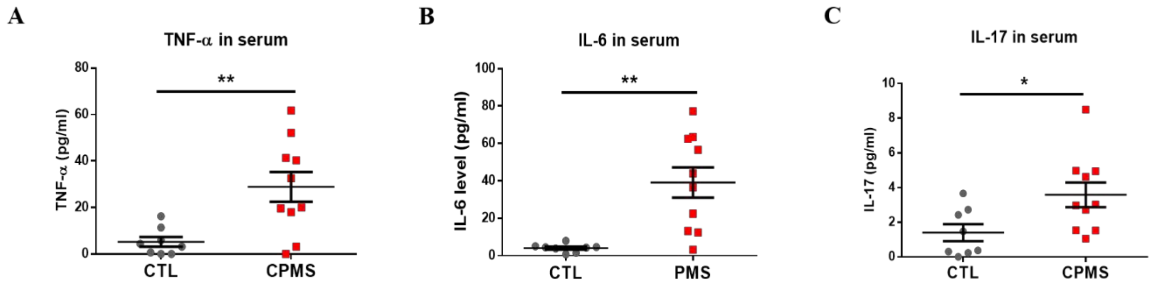

Fig.S5 Inflammatory cytokines in the serum of mice

Inflammatory cytokines were upregulated in serum of CPMS mice. (A) TNF-α (CTL (n=8); 5.214 ± 2.073, CPMS (n=10); 28.89 ± 6.405), (B) IL-6 (CTL (n=8); 4.071 ± 0.7785, CPMS (n=10); 39.15 ± 8.083), and (C) IL-17 (CTL (n=8); 1.406 ± 0.4906, 3.5900 ± 0.7087) were analyzed using ELISA. Unpaired t-test was used for analysis in between groups.

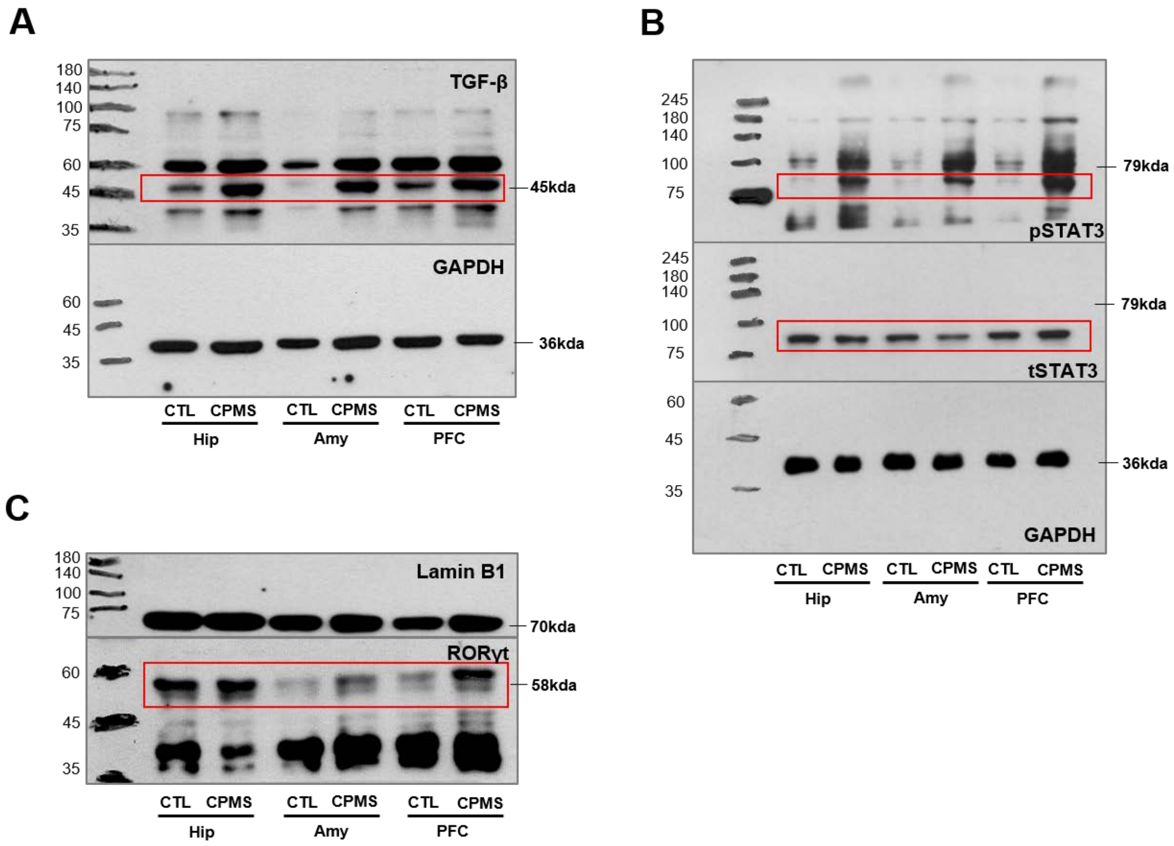

Fig.S6 The full blots of Fig.4

(A) The band in the red box was read for quantification. (B) TGF-β, (C) pSTAT3/tSTAT3 in the cytosol were normalized by GAPDH. (D) RORγt in the nucleus was normalized by Lamin B1. An unpaired t-test was used for analysis in each region.

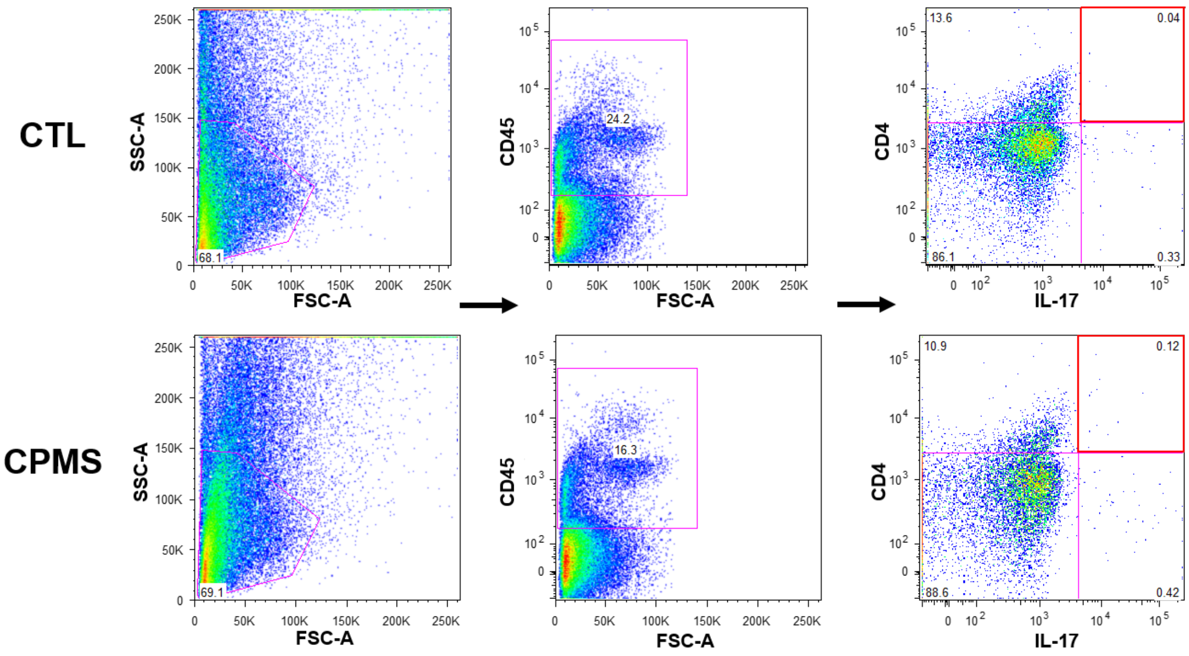

Fig.S7 The population of Th17 cells

Flow cytometry plots demonstrating the gating strategy used to identify the brain Th17 cell population. (A) Whole-brain cells were discriminated against by the plotting side scatter area (SSC-A) against the forward scatter area (FSC-A). (B) Following gated by CD45, (C) population of $CD45^{+}CD4^{+}IL17^{+}$ (Th17 cells) was measured in the red box. An unpaired t-test was used for analysis in each region.

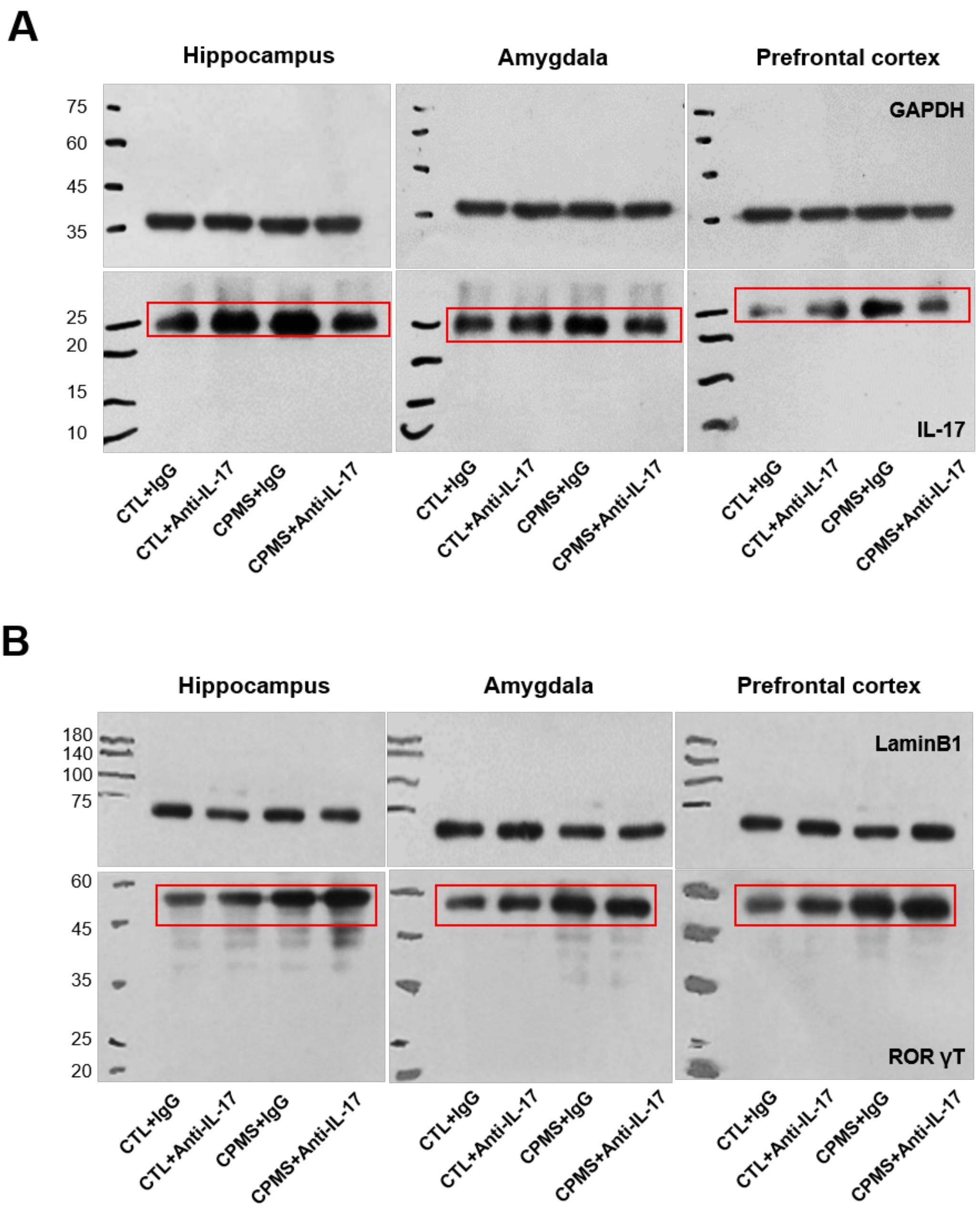

Fig.S8 The full blots of Fig.6

The band in the red box was read for quantification. (A) IL-17 in the cytosol were normalized by GAPDH. (B) RORγt in the nucleus was normalized by Lamin B1. An unpaired t-test was used for analysis in each region.

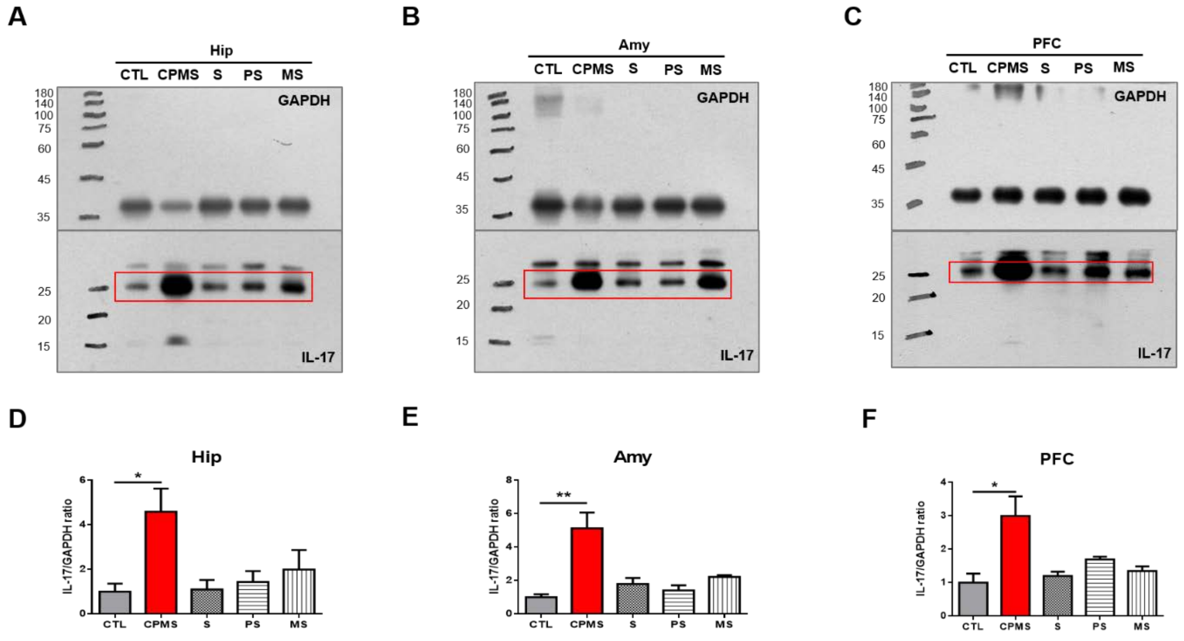

Fig.S9 The changes in IL-17 levels in the brain of mice

The band in the red box was read for quantification of IL-17 protein level in (A) Hip (Fig. 4G; $F_{(4,10)}$=4.597, *p*=0.0230), (B) Amy (Fig. 4H; $F_{(4,10)}$=11.65, *p*=0.0009), and (C) PFC (Fig. 4I; $F_{(4,10)}$=6.880, *p*=0.0063) of CPMS mice. Western blot analysis was performed using cytosol of (D) Hip (CPMS; 4.591 ± 1.040, S; 1.101 ± 0.4291, PS; 1.436 ± 0.4921, MS; 1.999 ± 0.8736 vs CTL), (E) Amy (CPMS; 5.133 ± 0.9420, S; 1.795 ± 0.3625, PS; 1.413 ± 0.3010, MS; 2.218 ± 0.1047 vs CTL), and (F) PFC (CPMS; 3.000 ± 0.5866, S; 1.201 ± 0.1288, PS; 1.694 ± 0.08339, MS; 1.348 ± 0.1407 vs CTL). All data are presented as mean ± SEM of three or more independent experiments. **p*<0.05, ***p*<0.01, ****p*<0.001, *****p*<0.0001 vs. CTL. IL-17 was normalized by GAPDH. One-way ANOVA was used for analysis between the groups.
